# Tetralogy of Fallot with restrictive ventricular septal defect by accessory tricuspid valve: repair with accessory valve preservation

**DOI:** 10.1186/s44215-023-00076-9

**Published:** 2023-10-02

**Authors:** Kenichi Amano, Yasutaka Hirata, Miyuki Shibata, Minoru Ono

**Affiliations:** 1 Faculty of Medicine, The University of Tokyo, Tokyo, Japan; 2 Department of Cardiac Surgery, The University of Tokyo Hospital, Tokyo, Japan

**Keywords:** Accessory tricuspid valve, Restrictive ventricular septal defect, Tetralogy of Fallot, Tricuspid regurgitation

## Abstract

**Background:**

Restrictive ventricular septal defect resulting in suprasystemic right ventricular pressure is a rare entity in tetralogy of Fallot patients. It is often caused by accessory tricuspid valve tissue protruding through the defect. Surgical resection of the accessory tricuspid valve has been recommended, but the risk of tricuspid regurgitation caused by the resection has not been widely discussed.

**Case presentation:**

A 5-month-old male who had been diagnosed with tetralogy of Fallot was referred for progressive decline in oxygen saturation. Preoperative echocardiogram revealed restrictive ventricular septal defect and severe pulmonary stenosis, suggesting suprasystemic right ventricular pressure (> 160 mmHg). Intraoperatively, accessory tricuspid valve tissue was found to be attached to the superior edge of the ventricular septal defect and partially occlude the aortic valve. The risk of tricuspid regurgitation caused by accessory tricuspid valve resection was high due to the complicated relationship between the true tricuspid valve and the accessory tricuspid valve. Therefore, the accessory tricuspid valve was preserved on the right ventricular side of the ventricular septum to avoid the risk of tricuspid regurgitation. Postoperative echocardiogram did not show tricuspid regurgitation or left ventricular outflow tract obstruction, and the patient was discharged to home on postoperative day 35 without major complications.

**Conclusions:**

We herein reported a high-risk patient of tetralogy of Fallot with restrictive ventricular septal defect caused by accessory tricuspid valve who successfully underwent definitive repair with accessory tricuspid valve preservation considering the risk of tricuspid regurgitation.

**Supplementary Information:**

The online version contains supplementary material available at 10.1186/s44215-023-00076-9.

## Background

Tetralogy of Fallot (TOF) is usually characterized by nonrestrictive ventricular septal defect (VSD). In rare cases, accessory tricuspid valve prolapses through the VSD, resulting in restriction of the defect size and suprasystemic right ventricular pressure [1–6]. Due to the high mortality of such cases, early surgical correction is recommended to prevent right ventricular failure [2–5]. It has been recommended that mobile accessory valve be resected since it may cause left ventricular outflow tract (LVOT) obstruction if it is remained on the left ventricular aspect of the patch [1]. However, the risk of tricuspid regurgitation (TR) caused by accessory tricuspid valve resection has not been widely discussed. Herein, we present a case of TOF with restrictive VSD caused by accessory tricuspid valve, which was preserved to avoid the risk of TR. We highlight a safer management of the accessory tricuspid valve to reduce the risk of postoperative complications in such high-risk patients.

## Case presentation

**Fig. 1:**
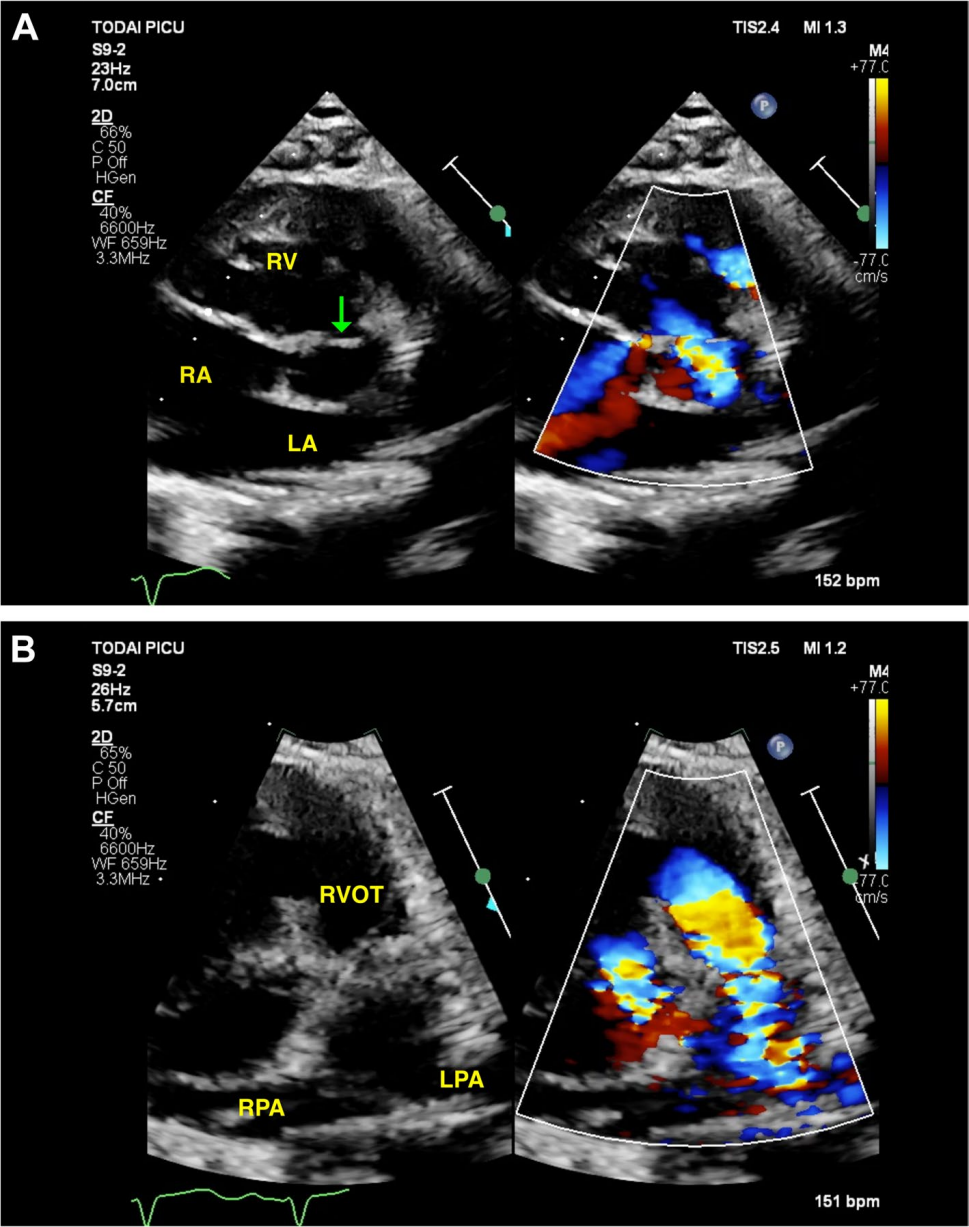
Preoperative Doppler echocardiography. **A** Parasternal short-axis view showed restrictive ventricular septal defect (VSD) caused by flap-like tissue in continuity with the tricuspid valve (green arrow), that is, accessory tricuspid valve, with right-to-left shunt and with a gradient of 61 mmHg, suggesting suprasystemic right ventricular pressure. **B** Parasternal short-axis view showed severe pulmonary stenosis with a peak right ventricular outflow tract (RVOT) gradient of 149 mmHg, which also suggested extremely high right ventricular pressure

**Fig. 2:**
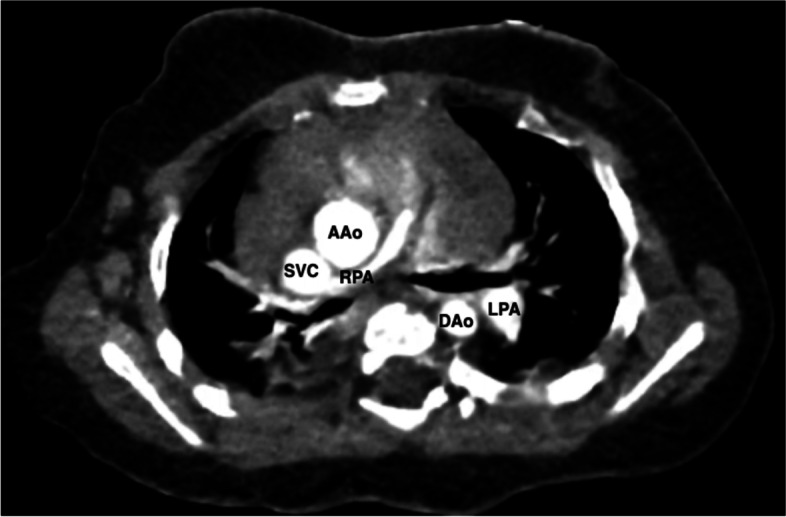
Preoperative computed tomography (CT). Axial images showed a hypoplastic right pulmonary artery (RPA) with a diameter of 3.8 mm

A 5-month-old male who had been diagnosed with TOF was referred for progressive decline in oxygen saturation. He had no history of cyanotic spells, but systemic oxygen saturation gradually dropped to 80%. The echocardiogram revealed restrictive VSD caused by flap-like tissue in continuity with the tricuspid valve and moving towards the VSD in systole with right-to-left shunt with a gradient of 61 mmHg, suggesting suprasystemic right ventricular pressure (Fig. 1A). It also demonstrated severe pulmonary valvular and supravalvular stenosis with peak right ventricular outflow tract (RVOT) gradient of 149 mmHg (Fig. 1B). Right ventricular pressure was estimated to be over 160 mmHg. There was mild TR. Contrast-enhanced computed tomography (CT) demonstrated a hypoplastic right pulmonary artery with a diameter of 3.8 mm (Fig. 2). Left pulmonary artery was relatively large with a diameter of 7.8 mm, although the proximal origin of the left pulmonary artery was slightly narrow. These findings suggested that early decompression of the right ventricle was necessary to prevent subsequent right ventricular failure.

**Fig. 3:**
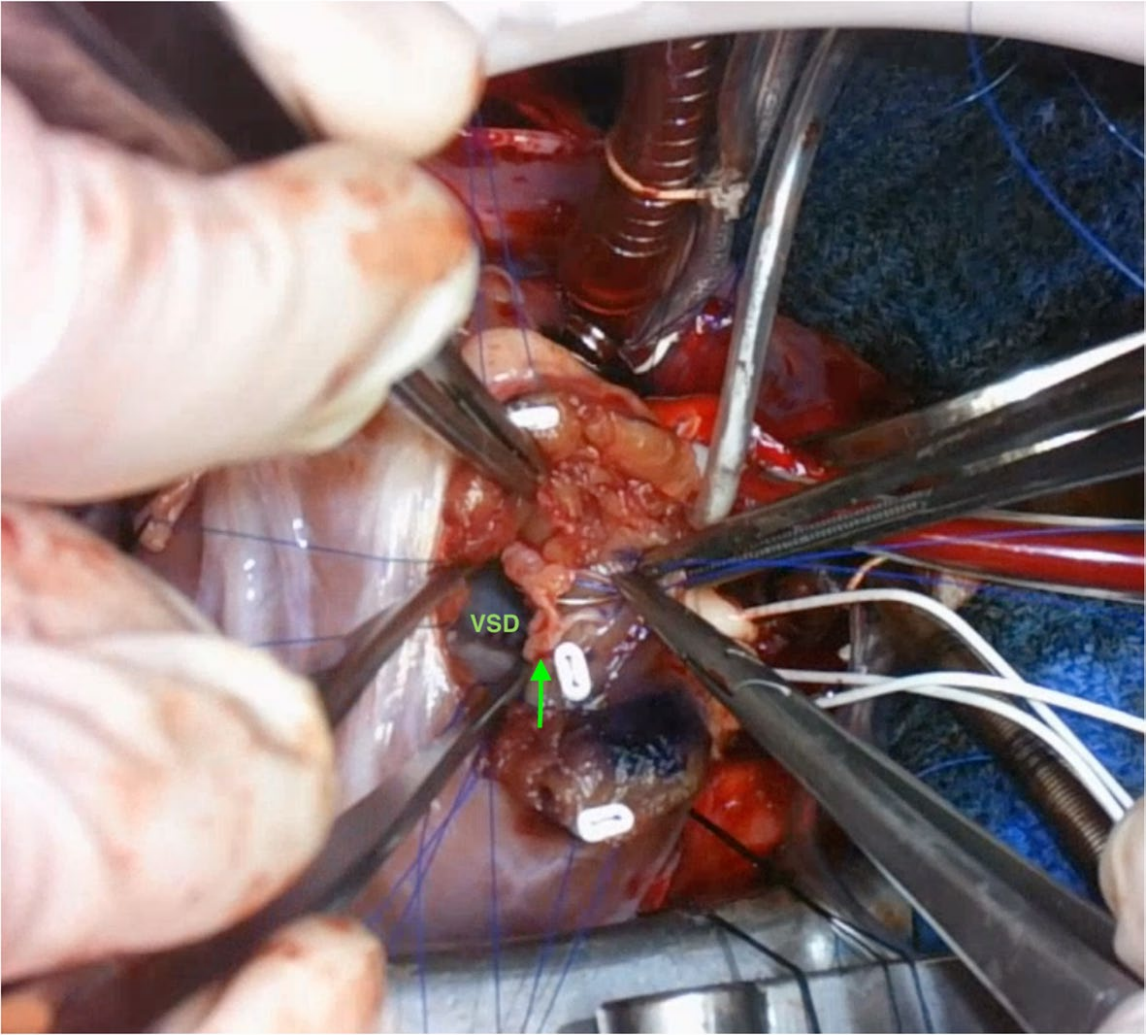
Operative findings. When the right ventricular outflow tract (RVOT) incision was performed, accessory tricuspid valve tissue (green arrow) was found to be attached to the anterosuperior edge of the ventricular septal defect (VSD), thereby restricting the defect, which was completely different from the usual attachment of the tricuspid valve. The location of tacking the accessory tissue, which was retracted and used as anchorage tissue for the placement of pledgeted sutures, is also shown

Intraoperatively, the pulmonary annulus was found to be diminutive, and transannular patch repair was planned. After RVOT incision, accessory tricuspid valve was found to be attached to the superior edge of the VSD and partially occlude the aortic valve (Fig. 3). Resection of the accessory tricuspid valve was considered, but the relationship between the true tricuspid valve and the accessory valve was complicated. Therefore, it was assumed that leaving the accessory tricuspid valve on the right ventricular side is the safest way to avoid postoperative TR. The accessory tricuspid valve was attached to the anterosuperior edge of the VSD, which was completely different from the usual attachment of the tricuspid valve. The VSD was closed with an expanded polytetrafluoroethylene (ePTFE) patch with pledgeted sutures, using the retracted accessory tricuspid valve as anchorage tissue for the placement of sutures (also see Additional file 1). RVOT was augmented with transannular patch, and competency of the tricuspid valve was confirmed by a water injection test. The right pulmonary artery was reconstructed with autologous pericardium.

Postoperative echocardiogram showed normal right ventricular function and no TR, residual VSD shunt, LVOT obstruction, nor RVOT and pulmonary artery stenosis. Although the patient underwent pericardial drainage for chylous pericardial effusion on postoperative day 14, he was discharged to home on postoperative day 35 without any other complications. The patient remained asymptomatic at 9-month follow-up.

## Discussion and conclusions

Restrictive VSD, resulting in suprasystemic right ventricular pressure, is an uncommon anomaly in TOF patients [1–6]. Flanagan and associates described the echocardiographic and anatomic features in four patients (1.48%) with restrictive VSD among 269 patients with TOF undergoing surgical repair [3]. In the majority of such cases, restrictive VSD is caused by accessory tricuspid valve protruding through the defect [1–6]. Suprasystemic right ventricular pressure caused by restrictive VSD represents a major stimulus for excessive right ventricular hypertrophy and is associated with increased surgical mortality [2–6]. Therefore, early surgical decompression of the right ventricle is important to prevent right ventricular failure resulting from right ventricular pressure overload [2–6].

It is also important to recognize the precise morphology of the accessory tricuspid valve preoperatively and intraoperatively. Faggian and associates described two types of accessory valve, the mobile type and the fixed type [1]. It has been recommended that mobile accessory valve be resected since it may result in LVOT obstruction if it is remained on the left ventricular aspect of the patch at the end of the procedure [1]. Yoshimura and associates reported six cases of TOF with accessory tricuspid valve, and in five cases, it was found to be attached to the posterior edge of the VSD and excised successfully [7].

In our case, however, the accessory tricuspid valve was connected with the true tricuspid valve and attached to the superior edge of the VSD. It was feared that resection of the accessory tricuspid valve might accidentally cause partial resection of the true tricuspid valve tissue, resulting in severe TR. We estimated that leaving the accessory tricuspid valve in situ would not trigger severe problems since preoperative echocardiogram showed only mild TR. Therefore, the accessory tricuspid valve was retracted and left on the right ventricular side of the ventricular septum so as not to cause LVOT obstruction and accidental TR. Although it appears ideal to resect the accessory tricuspid valve completely as suggested in a previous report [1], in some cases, it might be preferable to choose the easier and safer option to reduce the risk of postoperative complications.

## Supplementary Information

**Additional file 1. **Surgical video. The accessory tricuspid valve was left in situ on the right ventricular side of the ventricular septum because the risk of tricuspid regurgitation caused by accessory tricuspid valve resection was high. When closing the ventricular septal defect with an expanded polytetrafluoroethylene patch, the accessory tricuspid valve was retracted and used as anchorage tissue for the placement of pledgeted sutures.

## Data Availability

The datasets used for this case report are available from the corresponding author upon reasonable request.

## Abbreviations

AAo: Ascending aorta
CT: Computed tomography
DAo: Descending aorta
ePTFE: Expanded polytetrafluoroethylene
LA: Left atrium
LPA: Left pulmonary artery
LVOT: Left ventricular outflow tract
RA: Right atrium
RPA: Right pulmonary artery
RV: Right ventricle
RVOT: Right ventricular outflow tract
SVC: Superior vena cava
TOF: Tetralogy of Fallot
TR: Tricuspid regurgitation
VSD: Ventricular septal defect
